# Cotton proteomics: Dissecting the stress response mechanisms in cotton

**DOI:** 10.3389/fpls.2022.1035801

**Published:** 2022-11-17

**Authors:** George Bawa, Zhixin Liu, Yaping Zhou, Shuli Fan, Qifeng Ma, David T. Tissue, Xuwu Sun

**Affiliations:** ^1^ State Key Laboratory of Cotton Biology, Key Laboratory of Plant Stress Biology, School of Life Sciences, Henan University, Kaifeng, China; ^2^ State Key Laboratory of Cotton Biology, Institute of Cotton Research, Chinese Academy of Agricultural Sciences (ICR, CAAS), Anyang, China; ^3^ Hawkesbury Institute for the Environment, Western Sydney University, Richmond, NSW, Australia

**Keywords:** adaptation, cotton, environmental stress, fiber development, proteomics

## Abstract

The natural environment of plants comprises a complex set of biotic and abiotic stresses, and plant responses to these stresses are complex as well. Plant proteomics approaches have significantly revealed dynamic changes in plant proteome responses to stress and developmental processes. Thus, we reviewed the recent advances in cotton proteomics research under changing environmental conditions, considering the progress and challenging factors. Finally, we highlight how single-cell proteomics is revolutionizing plant research at the proteomics level. We envision that future cotton proteomics research at the single-cell level will provide a more complete understanding of cotton’s response to stresses.

## Introduction

**Figure 1:**
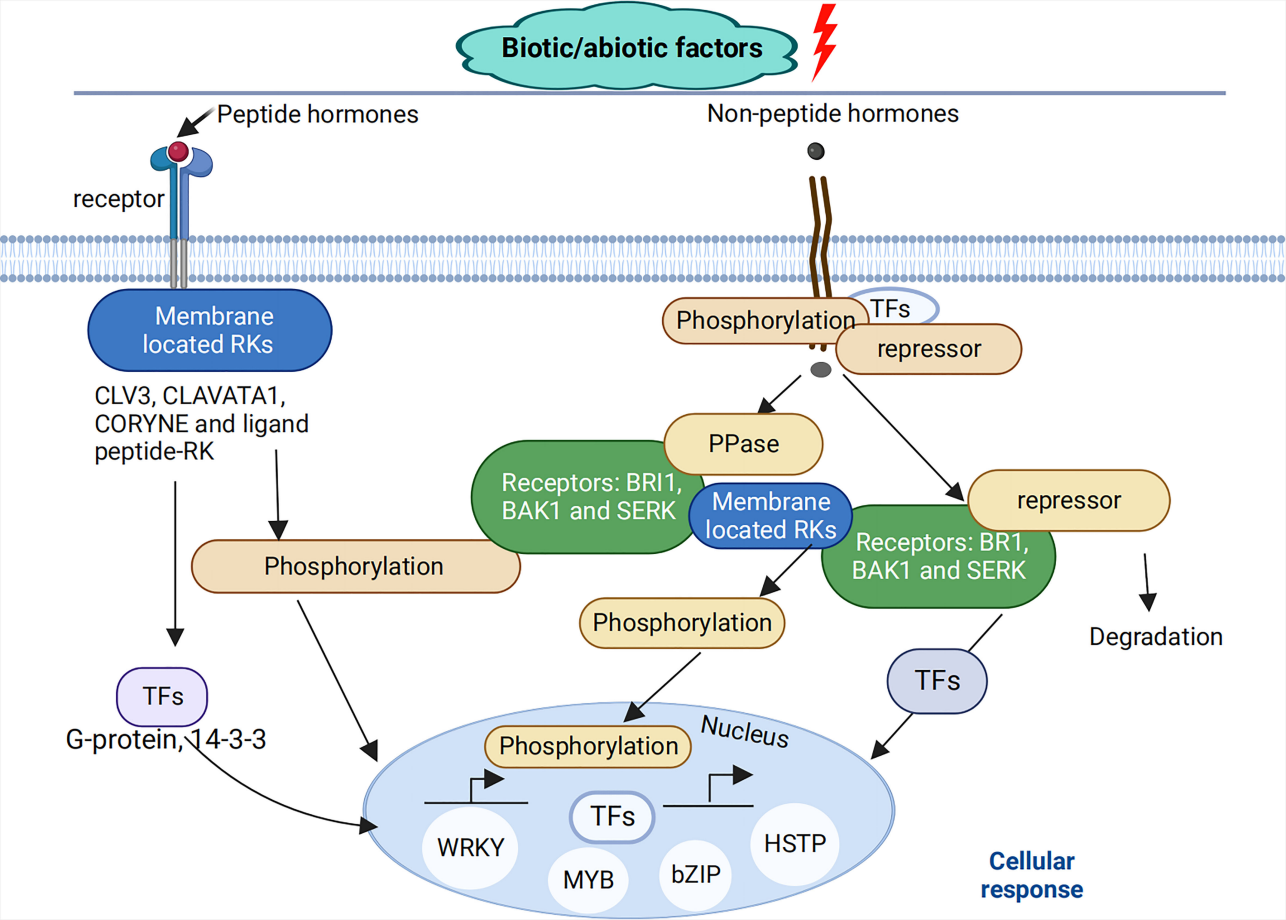
Plant cellular signaling cascades. Throughout their developmental period, plants are attacked by different biotic and abiotic stresses. These stress signals are recognized by membrane-located RKs, which play an important role in plant signaling pathways either through peptide hormones such as CLAVATA3, CLAVATA1, CORYNE and other ligand peptides –RK interactions or non-peptide hormones, such as a membrane-bound receptor named brassinosteroid-insensitive 1 (BRI1), which interacts with BCL2 antagonist/killer1 (BAK1), and somatic embryogenesis receptor-like kinase (SERK) shown to be involved in different signaling pathways under stress conditions. These transmembrane receptor-like kinases transmit signals through the plasma membrane, which activates a signaling cascade related to post-translational modifications of proteins, with signals activating the expression of transcription factors (TFs), such as myb-related protein (MYP), basic leucine zipper (bZIP), heat stress transcription factor (HSTP), WRKY, etc., as a form of response to these stresses.

Cotton (*Gossypium* spp.) is an essential industrial crop cultivated throughout the world for the production of textile fiber and cottonseed oil (Li et al., 2007; Santhosh and Yohan, 2019; Zhao et al., 2022). However, stress conditions often affect cotton growth and development, thus decreasing cotton yield. Over the past decade, cotton yield and quality have been decreased by different abiotic stresses such as drought, shade, and temperature (Wang et al., 2014; Umbetaev et al., 2015; Ullah et al., 2016; Guo et al., 2017; Li et al., 2020) and biotic stress such as fungal infections (Gao et al., 2013; Zhang T. et al., 2016; Zhang et al., 2017). As part of evolution, cotton plants have evolved several defense mechanisms that generate a rapid response to incoming stresses, enhancing tolerance to combat these unfavorable environmental factors (Wang et al., 2014; Guo et al., 2017; Kerry et al., 2018; Bawa et al., 2019; Zhou et al., 2019; Li et al., 2020; Bhat et al., 2022). Stress signals are recognized by plasma membrane or intracellular receptors, which results in the activation of a signaling cascade related to post-translational modifications of the proteins, with signals transduced to transcription factors (TFs), thus activating transcriptional responses (**Figure 1**), suggesting that knowledge of cotton gene and protein identification, function, and expression pattern under stress conditions is essential for increasing cotton yield (Wang et al., 2012; Zhang et al., 2017; Nagamalla et al., 2021).

Recent knowledge has shown how transcriptome analysis has revealed the functions of a large number of stress-responsive genes in cotton (Han et al., 2019; Zhu H. et al., 2021). However, the up-regulated proteins and mRNA activities often do not correspond to each other as a result of post-translational activities (Pradet-Balade et al., 2001), which suggests that genome and transcriptome findings alone cannot be used to determine plant gene function and the regulatory mechanisms of plants under stress conditions. Meanwhile, other studies have shown that plant response to changing environmental conditions is directly linked with the upregulation of defense-related proteins (Kerry et al., 2018; Liu L. et al., 2019; Sinha et al., 2021), which means that proteomics could provide mechanistic insights into the function of differently expressed proteins during cotton stress acclimation (Chen et al., 2020) and developmental processes (Zhu et al., 2018). The term “proteome” refers to the protein component of a given sample (organisms or plants), while “proteomics” refers to the quantification and identification of these proteins (Wilkins et al., 1996). Plant research uses proteomics approaches to understand plant growth dynamics and how plants respond to stress conditions to improve crop tolerance mechanisms, which increases crop yield and quality in our agricultural systems. In recent times, single-cell proteomics profiling has been used to study protein dynamics in plants. Single-cell proteomics allows the identification of many proteins expressed within thousands of individual cells at a given time (Clark et al., 2022). Single-cell-type proteomics treats biological samples as heterogeneous, which reveals the actual functions of cells in plant developmental processes (Dai and Chen, 2012; Potts et al., 2022). Recent breakthroughs in single-cell proteomics have enabled us to distinguish different cellular subpopulations through large-scale protein profiling (Clark et al., 2022). Hence, considering the constant regulation of cotton growth and development by different stress conditions, this review discusses the progress in cotton proteomics research. More importantly, we highlight how single-cell proteomics could revolutionize plant response to stress conditions in the coming years.

## Cotton proteomics approaches

Selecting a methodology for separating and identifying plant proteins is an important step to consider in plant proteomics analysis. A reliable analytical resolution in the separation and identification steps is required for a complete or successful extraction process. In response to stress, cotton plants activate defense genes to enhance tolerance through changes in defense protein expression levels (Wang et al., 2012; Tu et al., 2017). However, the regulation of gene expression in plants is controlled by several signal-sensing networks of phosphorylation and dephosphorylation activity (Abreu et al., 2013), which suggests that the application of proteomics at the cotton stress response level could assist in identifying key defense proteins involved in a particular stress condition. The cotton proteomic analysis comprises either gel-based method (protein separation using gel electrophoresis, quantification, spot digestion, and mass spectrometric analysis) or gel-free based method (protease breakdown of protein samples and liquid chromatographic separation and spectrometric analysis) (Champagne and Boutry, 2013). Despite the high labor and time-consuming nature of the two-dimensional gel electrophoreses (2-DE) approach, several developmental studies have used the technique for cotton protein quantification and separation (Coumans et al., 2009; Rabilloud and Lelong, 2011; Zhou et al., 2014; Li et al., 2015) (**Table 1**). The cotton gel-based technologies include 2-DE at the separation level and mass spectrometry (MS) at the identification level (Wang et al., 2011), which have been reviewed in cotton proteomic analysis (Zhou et al., 2014). In the 2-DE analysis, the protein spots are often stained with Coomassie brilliant blue and fluorescent dye (Chevalier et al., 2004). Using advanced mass spectrometry, the 2-DE analysis enhances different proteins characterized in a single gel (Magdeldin et al., 2014). These advantages of the 2-DE make it more applicable in post-translational modifications (PTMs) of cotton protein analysis (Zhou et al., 2014). Again, the 2-DE analysis is considered essential because of its increased identification and quantification of proteins with different expressions under different conditions and comparative expression of protein complexes (O’Farrell, 1975; Heinemeyer et al., 2009; Rabilloud, 2012). As a result of its reliability, 2-DE has been used to effectively characterize cotton organelles and other tissues, including cotton leaf and root proteomics, successively (Coumans et al., 2009; Pang et al., 2010; Meng et al., 2011). To obtain higher protein spots in cotton, Yao et al. (2006) added polyvinylpolypyrrolidone (PVPP) into cotton grinding samples to remove unwanted compounds such as polyphenols and lipids. They also added 80% cold acetone in water to prevent protein pellets from lipid contamination. Further, cold acetone was used to clean the tissue powder while suspended in an extraction buffer to enhance extraction ability and supplemented with 2% SDS to promote the solubility of proteins, making this an efficient protocol for cotton protein extraction. However, cotton protein analysis with the 2-DE gel approach can sometimes be constrained by the sensitivity, linearity, and homogeneity of the staining processes and is in line with mass spectrometry. Protein identification using fluorescent dyes can sometimes be problematic since it combines sensitivity and compatibility with mass spectrometry techniques (Rabilloud and Lelong, 2011). Another constraint of the 2-DE gel analysis is its low-level identification of low abundant proteins (Rabilloud and Lelong, 2011). Again, the 2-DE approach can only separate up to about 30–50% of a tissue proteome and often cannot separate all the proteins in certain complex cotton tissues (Yao et al., 2006). The above-listed constraints of the 2-DE gel approach led to the development of gel-free proteomics technologies applied to cotton.

**Table 1:** Cotton proteomics studies according to stress type, tissue and method used.

Stress type	Organ/Tissue	Method	References
Cadmium stress	Leaves	2-DE	Daud et al. (2015)
Drought	Leaves	2-DE	Deeba et al. (2012)
Drought	Root	Tandem Mass Tag-based (TMT)	Xiao et al. (2020)
Drought	Root	2-DE	Zhang H. et al. (2016)
Nitrogen stress	Fiber	2-DE	Wang et al. (2012)
Low temperature	Fiber	2-DE	Zheng et al. (2012)
Fungal infection	Root	2-DE	Wang et al. (2011)
Fungal infection	Root	2-DE	Coumans et al. (2009)
Fungal infection	Root	2-DE	Zhao et al. (2012)
Fungal infection	Root	iTRAQ	Zhang et al. (2017)
Salinity Low light	Root Fiber	iTRAQ 2-DE	Li et al. (2015) Hu et al. (2017)

**Figure 2:**
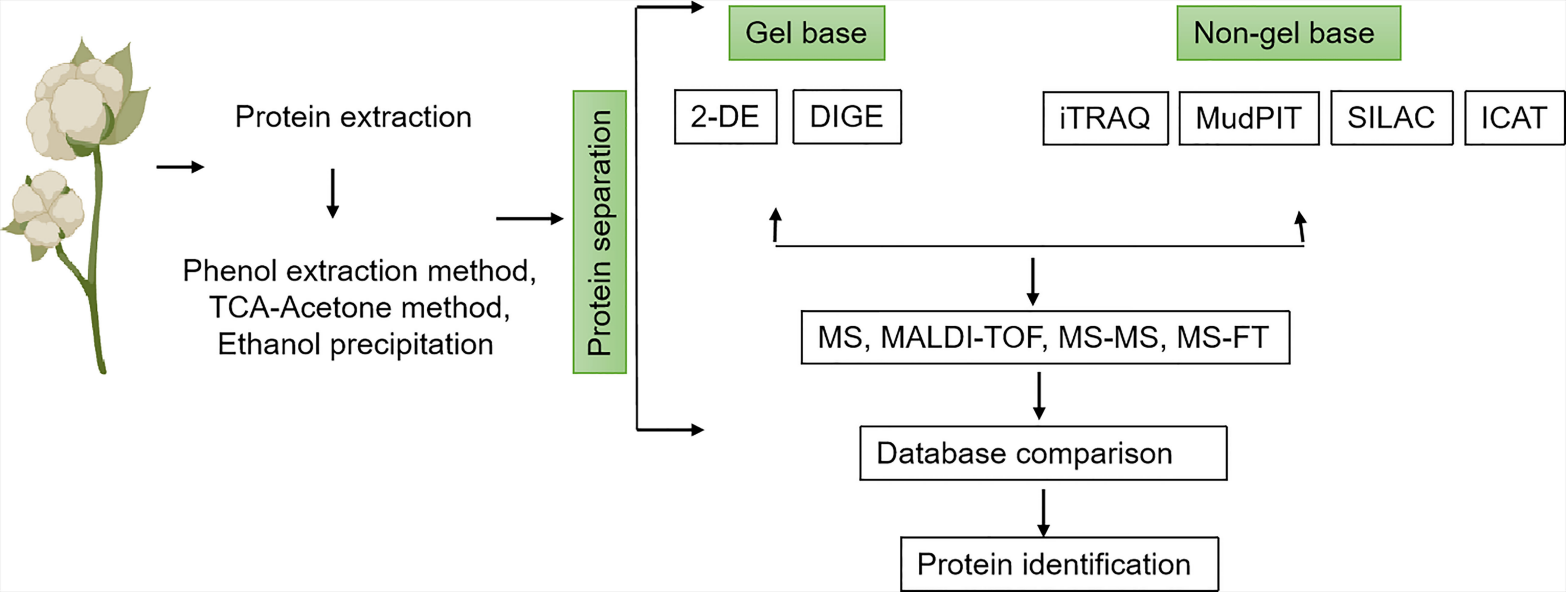
Schematic workflow of the different methods used in cotton proteomics analysis. Proteins are extracted from tissues of interest using any of the following methods: phenol extraction, TCA-Acetone, or ethanol precipitation. This is followed by protein separation either using a gel base (2-DE or DIGE) or a non-gel base (iTRAQ, MupPIT, SILAC, ICAT) method. Proteins can further be analyzed by MS, MALDI-TOF, MS-MS, or MS-TF. After analysis, data comparison is performed for final protein identification.

In addition to the 2-DE gel-based approach, various sophisticated gel-free proteomic techniques have also been exploited in cotton proteomic analysis, which suggests a growing level in the field of differential proteomics (**Figure 2**). The gel-free proteomic analysis has the ability to overcome certain challenges of the 2-DE gel approach, such as detection sensitivity, low-level detection of hydrophobic proteins, and high throughput worldwide proteome analysis of complex biological systems. The gel-free technique includes tag-based labeling, metabolic labeling, and label-free techniques. With tag labeling, various mass tags like ICAT, iTRAQ, TMT, and dimethyl labeling are introduced into the proteins, while the metabolic labeling techniques include SILAC and ^15^N labeling (Riter et al., 2011). Various studies have shown that these gel-free approaches are more reproducible and reduce biases more effectively than the 2-DE gel method (Lee et al., 2010). A study conducted by Nouri and Komatsu (2010) investigated a proteomic analysis of soybean plasma membrane under osmotic stress with 4 and 8 protein spots shown as high and low abundance proteins, respectively, using the 2-DE gel technique, while 11 and 75 proteins were observed as high and low abundance proteins using nanoLC-MS. Using the same comparative method, Van Cutsem et al. (2011) extracted 680 and 850 proteins from *Nicotiana tabacum* trichomes via the 2-DE gel technique and gel-free method, respectively, which highlights the comparative advantage of the gel-free protein analysis over the gel-based method. However, despite the numerous advantages of the gel-free-based technique over the 2-DE gel approach, the gel-free technique has some challenges, limiting its application in cotton proteomics research in many laboratories. In the gel-free-based technique, peptides found in multiple proteins reduce the reliability of identified proteins, and the cost of this technology makes it more expensive for cotton proteomics analysis, thus limiting plant research progress. Nevertheless, the 2-DE gel-based approach is commonly used alongside the mass-spectrometry technique for cotton proteomics analysis despite the substantial progress in other proteomics methods (Nouri and Komatsu, 2010; Van Cutsem et al., 2011), especially when dealing with several quantification comparison samples, and the lower cost of this technology makes its application easier and affordable for many cotton research laboratories (**Table 1**).

## Proteomics and stress adaptation in cotton

In their natural environments, biotic or abiotic stresses negatively regulate plant growth and development (Guo et al., 2019; Tang et al., 2019; Wu and Li, 2019; Li et al., 2020; Zhang et al., 2021; Liu et al., 2022B; Mostofa et al., 2022), which activates plant stress defense mechanisms (Sun et al., 2011; Shabala et al., 2014; Chen et al., 2017; Guo et al., 2019; Wu and Li, 2019; Li et al., 2020; Li et al., 2021; Lv et al., 2021; Zhang et al., 2021; Chieppa et al., 2022; Liu et al., 2022a; Solis et al., 2022; Wu et al., 2022) (**Figure 1**). Likewise, in the life of the most prestigious industrial crop, cotton growth and development is often regulated by different stress conditions that initiate several defense mechanisms at the physiological, cellular, and molecular levels, which include a change in plant height and leaf size, upregulation of antioxidant defense enzymes, and increase in the levels of defense-related genes and proteins (Nagamalla et al., 2021; Qamer et al., 2021). Since cotton’s genomic sequence is already available, post-transcriptional investigations will positively impact cotton growth and development by understanding the regulatory mechanisms underlying how cotton plants respond to these stresses. Since its introduction into plant research, proteomics, an “omic” approach that enhances the quantification and identification of differently expressed proteins, has enabled the identification of post-transcriptionally related proteins, which play a key role in plant response to stress. Biotic and abiotic stresses induce changes in protein expression in cotton, and using proteomics techniques provides information and understanding of the functions of the key proteins expressed under stress conditions (Gao et al., 2013; Liu L. et al., 2019). Thus, breeders can use the identification of these stress-responsive proteins to develop stress-tolerant cotton varieties. In addition to elucidating the functions of cotton proteins, proteomics also enhances our understanding of phenotypic variations during cotton stress adaptation processes. (**Table 1**) describes how cotton tissues or organs respond to stress conditions using various proteomics methods. In the case of abiotic stress, throughout cotton-growing areas globally, cotton growth and development have been affected by an increasing number of abiotic stresses, which negatively regulate cotton’s physiological development (Wang et al., 2014; Snider et al., 2021). For instance, a study by Zheng et al. (2017) using iTRAQ-based quantitative proteomic analysis to analyze the mechanism involved in induced premature leaf senescence in two cotton genotypes under cold conditions showed 443 differential abundant proteins (DAPs) were identified from high-confidence proteins at four different stages between premature cotton and non-premature cotton genotypes, with 158 proteins being over-accumulated, 238 proteins down-regulated, and 47 proteins showing overlapped accumulation in all the different stages. The Gene Ontology enrichment analysis showed that cold-responsive and hormonal-related genes were more highly accumulated in the premature genotype than in the non-premature genotype. Significantly, 58 proteins were involved in abiotic stress, hormonal signaling, and leaf greenness regulation, consisting of 26 cold-responsive proteins (**Table 2**). Together, this study demonstrated that changes in plant leaf development undergo several differential protein expressions, which require identification and functional classification using proteomics approaches. In addition, Nagamalla et al. (2021) investigated the molecular mechanisms underlying drought tolerance of two cotton genotypes, *Bacillus thuringiensis* cotton and hybrid cotton, using 2DE-DIGE proteomics analysis. It was observed in this study that 509 and 337 different proteins were expressed in *Bacillus thuringiensis* and the hybrid genotype, respectively, compared to their controls. Interestingly, the transcript analysis performed alongside the identified drought-related proteins confirmed a significant correlation in expression. *In silico* analysis of the differentially expressed proteins ATPase ß subunit (ATPB), nucleobase-ascorbate transporter 9 (NAT9), early responsive to dehydration (ERD), late embryogenesis abundant (LEA) proteins, and embryo-defective 2001 (EMB2001) proteins were correlated with different drought-related genes such as late embryogenesis abundant (LEA), APETALA2/Ethylene Responsive Factor (AP2/ERF), WRKY, and neuronally altered carbohydrate (NAC). These different proteins played an important role in cotton drought response, especially in the *Bacillus thuringiensis* genotype. The significant drought response in the *Bacillus thuringiensis* genotype induced overexpression of photosynthetic proteins, which elevated lipid metabolism, induced cellular detoxification, decreased biosynthesis of unwanted proteins, improved stomatal functioning, and increased antioxidant activity such as catalase (CAT), superoxide dismutase (SOD), peroxidase (POD), and ascorbate peroxidase (APX) compared to the hybrid genotype, suggesting that proteomics technologies may provide a better understanding of cotton’s physiological response under drought stress, which could help in developing drought-tolerant and high-yielding cotton genotypes.

**Table 2:** List of proteins and related functions.

Stress type	Total proteins	Up-regulated proteins	Down-regulated proteins	Function	References
Drought	110			Cellular structure, antioxidants, and metabolism.	Zhang et al. (2017)
Low temperature	37			Soluble sugar metabolism, cell wall loosening, cellular response, cellulose synthesis, cytoskeleton, and redox homeostasis.	Zheng et al. (2012)
Fungal infection	68	51	17	Stress defense, metabolism, and lipid biosynthesis.	Wang et al. (2011)
Fungal infection	174			ROS metabolism, induction of various histone-modifying, and DNA methylating.	Zheng et al. (2017)
Fungal infection	188			Stimulus-response, cellular and metabolic processes.	Gao et al. (2013)
Low light	49			39 proteins were involved in signal transduction, energy metabolism, cytoskeleton, nitrogen metabolism, and stress response.	Hu et al. (2017)
Leaf senescence	195	91	104	Nitrogen metabolism, photosynthetic, and diterpenoid biosynthesis.	Liu L. et al. (2019)
Dwarfism	687			Catalytic, binding, and transporter-related activity.	Tu et al. (2017)

Similar to abiotic stress, biotic stress also regulates several physiological activities in cotton by introducing destructive pathogens at the growth stage. For example, Zhang et al. (2017) used an iTRAQ-based proteomic method to understand cotton pathogen interaction to further investigate pathogenic-related proteins involved in cotton’s disease resistance or tolerance. In this study, a total of 174 differentially induced proteins were observed in cotton plants as a result of *R. solani* infection (**Table 2**). These differentially induced proteins played a significant role in reactive oxygen species (ROS) metabolism and induction of various histone-modifying and DNA-methylating proteins resulting from *R. solani* infection, suggesting that the redox homeostasis and epigenetic regulation were vital for cotton’s resistance against *R. solani* infection. Further changes in phenylpropanoid biosynthesis-related protein expression in response to *R. solani* infection suggest a significant contribution of secondary metabolic activity in response to fungal infection in cotton. This study showed that the induction of different innate immunity-related proteins significantly contributes to cotton’s resistance to pathogen attacks. Verticillium wilt causes huge annual losses in cotton yield (Wang et al., 2016; Dadd-Daigle et al., 2021). Wang et al. (2011) demonstrated how different proteins are expressed in response to cotton and *Verticillium dahliae (V. dahliae)* interaction. This study conducted a comparative proteomic analysis between infected and non-infected cotton roots using 2-DE gel analysis. The findings showed that 51 up-regulated and 17 down-regulated proteins were involved in stress defense, metabolism, and lipid biosynthesis. Importantly, it was observed that ethylene defense signaling and biosynthesis were induced in cotton roots due to *V. dahliae* infection. It was also observed that the Bet v 1 family proteins were possibly involved in cotton’s defense against *V. dahliae* infection (**Table 2**). Gao and colleagues performed a comparative proteomics analysis to further understand the mechanisms of cotton’s resistance to *V. dahliae* (Gao et al., 2013). The study uncovered 188 differentially expressed proteins by matrix-assisted laser desorption ionization time-of-flight/time-of-flight (MALDI-TOF/TOF) mass spectrometry analysis and classified them into 17 biological functional groups based on Gene Ontology annotation. Several of these proteins were related to stimulus-response, cellular, and metabolic processes. The study further highlighted several genes involved in secondary metabolism, reactive oxygen burst, and salicylic acid (SA) signaling in cotton’s response to *V. dahliae* according to the analysis of GbSS12, a major regulator in the crosstalk between SA and jasmonic acid (JA) signaling pathways. In addition, three classes of genes involved in gossypol metabolism, brassinosteroids (BRs) signaling, and JA signaling were characterized using virus-induced gene silencing (VIGs). Continuously, the study revealed that gossypol, BRs, and JA act as major players in contributing to cotton’s resistance to *V. dahliae*, thus providing new insights into the molecular basis of cotton’s defense against *V. dahliae.* Together, these studies highlight the major role of proteomics analysis in dissecting the stress response mechanisms in cotton.

## Proteomics: For improving cotton fiber quality

Proteomics techniques are applied to farm animals to enhance the nutraceutical activity of the milk proteome or to check the *in vivo* performance of livestock animals (Bendixen et al., 2011; Roncada et al., 2012; D’Alessandro and Zolla, 2013). In the last decade, there has been increasing use of proteomics approaches in crop plants such as cotton to promote quality fiber and increase yield through improved breeding programs (Zhou et al., 2014; Ahmad, 2016; Liu et al., 2016). Cotton fiber is a widely used raw material in the textile industry. However, stress conditions often negatively regulate cotton fiber development, which decreases cotton fiber quality and yield.

As depicted in **Table 2**, several studies, including Zheng et al. (2012), used proteomics to show how low-temperature stress regulates protein expression during cotton fiber elongation using two cotton genotypes (low-temperature tolerant and low-temperature sensitive) planted at different sowing dates, which resulted in changes in environmental conditions. Proteomic investigations showed that a total of 37 proteins related to soluble sugar metabolism, cell wall loosening, cellular response, cellulose synthesis, cytoskeleton, and redox homeostasis were changed in response to the low-temperature stress according to the mass spectrometry identification, suggesting that the biosynthesis of these proteins was involved in the low-temperature tolerance of cotton fibers. This study’s results also show how proteomics approaches have significantly improved cotton fiber development. Low light is one of the most important environmental conditions reducing cotton yield in many cotton-growing areas (Pettigrew, 2001; Wang et al., 2005; Chen et al., 2014), suggesting that the identification of proteins involved in cotton’s response to low-light stress through proteomics has made a significant contribution to cotton fiber development. Using proteomic analysis, Hu et al. (2017) demonstrated how low-light conditions regulate cotton fiber elongation processes. The study showed that low-light stress decreased cotton fiber length. Proteomic analysis conducted at the four developmental stages (5, 10, and 15 days post-anthesis) indicated that 49 proteins were expressed under low light. Among these proteins, 39 were identified as well-known key low-light stress-responsive proteins significantly involved in signal transduction, energy metabolism, cytoskeleton structure, nitrogen (N) metabolism, and stress response. Moreover, the reduced fiber length in this study was linked with the levels of signal-related protein (phospholipase D), cytoskeletal proteins, carbohydrate metabolism proteins, and stress-responsive proteins down-regulated under low-light stress. These changes in protein levels in response to low light suggest that a further determination of the functions of all the identified proteins will go a long way to promoting cotton fiber development under low light. Changes in plant nutrient levels regulate plant growth and development (Ahmed M. et al., 2020; Shrivastav et al., 2020). For instance, N, phosphorus (P), and potassium (K) are required in large quantities and are limited in many soils. The deficiencies of macronutrients and micronutrients decrease cotton yield (Ahmed N. et al., 2020). Recently, Iqbal et al. (2022) demonstrated that low P tolerance in cotton is regulated by root morphology and physiology. The study showed that low P decreased dry matter, photosynthesis, and carbon metabolism in cotton, which could directly affect the yield.

Among these nutrients that highly regulate cotton fiber development is N (Read et al., 2006; Saleem et al., 2010; Wang et al., 2012; Snider et al., 2021; Van Der Sluijs, 2022). Using proteomics analysis, Wang et al. (2012) demonstrated how low N stress regulates cotton fiber elongation. The study used different N application rates: 0 kg hm^-2^ (N0), 240 kg hm^-2^ (N1), and 480 kg hm^-2^ (N2), equivalent to 0, 4.5, and 9.0 g per pot, respectively, where N0 represents N starvation, N1 normal N application, and N2 excess N application. The study showed that different nitrogen application rates regulate N biosynthesis in cotton fiber cells and fiber length, which revealed that cotton carbohydrate metabolism, antioxidants and hormonal, cell wall component synthesis, and amino acid metabolism-related proteins were significantly expressed during N stress (N0), with the carbohydrate metabolism proteins being the most expressed. Importantly, this study demonstrated that plants activate tolerance mechanisms such as expressing defense-related proteins for plant survival under stress conditions. Hence, the authors hypothesize that further functional analysis of the identified proteins could reveal the molecular mechanisms of cotton N tolerance for enhanced fiber quality. It can be concluded that understanding cotton fiber developmental changes under stress conditions using proteomics approaches will help decipher the molecular mechanisms governing stress tolerance in cotton, especially during fiber development.

## Proteomics: Toward physiological development of cotton

Proteomics technologies have been used to characterize proteome regulation throughout plant developmental processes. The plant growth process is constantly mediated by different stresses such as drought, temperature, and salinity (Afroz et al., 2011). Several studies have been conducted to understand how different proteins are expressed during plant physiological development (Tu et al., 2017; Zhu S. et al., 2021). Likewise, changes in cotton’s physiological activity during growth and development are regulated by changes in gene expression, which has a final consequence on protein levels and functions (Xu et al., 2013; Zhu S. et al., 2021). Proteomics techniques have been used to study proteome mediation during cotton developmental stages and different organ development. Various studies have been conducted to investigate the complete proteome profile of cotton during growth and development to understand the regulatory mechanisms underlying how proteomics approaches contribute to cotton stress tolerance mechanisms (Zhang Z. et al., 2016; Nagamalla et al., 2021). Here, we provide updates on how proteomics technologies contribute to certain physiological aspects of cotton’s developmental process. Using proteomics analysis, Zhang H. et al. (2016) determined the effect of different cotton genotypes on drought stress using 2-DE and MALDI-TOF mass spectrometry to analyze the proteome of two cotton genotypes (drought-sensitive and drought-tolerant) exposed to drought stress. A total of 110 protein spots were detected and identified as related to cellular structure, antioxidants, and metabolism. Other proteins such as ascorbate peroxidase, UDP-D- glucose pyrophosphorylase and DNA (cytosine-5) methyltransferase were significantly up-regulated in the drought-tolerant than in the sensitive genotype. Again, among the two genotypes, proteins such as translation initiation factor 5A and fungal-related proteins were in high abundance in the drought-tolerant, while ribosomal protein S12, cysteine, and actin were highly decreased in the drought-sensitive genotype. This enhances our understanding of how different proteins are induced in the roots of different cotton genotypes under drought stress.

Leaf senescence occurs in plants as the plant ages but sometimes can be induced by environmental stresses such as drought, temperature, shade, and salt (Munné-Bosch and Alegre, 2004; Brouwer et al., 2012; Liu L. et al., 2019), which involves the breakdown of intracellular organelles and macromolecules (Lim et al., 2007). One important growth stage that causes changes in cotton protein dynamics is leaf senescence (Liu L. et al., 2019). Using the iTRAQ method, Liu L. et al. (2019) characterized the protein expression patterns during the senescence of cotton leaves under field conditions. As part of the developmental processes, it was observed that the photosynthetic rates and photosynthetic pigment activities of the field-grown cotton were sharply decreased during the senescence period, which suggests that, as cotton ages, certain metabolic activities, including proteins, are broken down, which speeds up leaf yellowing (Liu L. et al., 2019). A total of 195 different proteins were identified by mass spectrometry, with 91 proteins being up-regulated and 104 down-regulated. In addition to changes in the protein dynamics, genes related to cotton photosynthetic biosynthesis, N metabolism, and diterpenoid biosynthesis expression levels significantly changed during the senescence process, which provides an interesting mechanism involved in proteome changes during cotton’s physiological development (Liu L. et al., 2019). The stem is a critical part of cotton that is regulated by different stress conditions. Tu et al. (2017) conducted a study using the iTRAQ approach to investigate the key elements and signaling pathways involved in cotton dwarfism using proteomic analysis. Two different cotton lines, dwarf line LA-1 and high near-isogenic line LH-1, were used for the study. It was observed that a total of 4849 proteins were identified from the two cotton lines, and 697 showed differential accumulations. Most DAPs had catalytic, binding, and transporter-related activity and were involved in the metabolism and processing (protein) pathways. A total of 7 DAPs that were mainly related to phytohormone (2-gibberellin, 3-cytokinin) receptors, cytokinin oxidase, and cytokinin-N-glucosyltransferase were increased in LA-1, while GA 20-oxidase was decreased in LH-1. The authors hypothesized that the DELLA-independent GA signaling pathway induced the dwarfism in LA-1 and suggested that the cytokinin-related element 1-2, gibberellin-insensitive dwarf, 3-ß-dioxygenase, and cytokinin oxidase could indicate dwarf cotton. The findings provide critical data for dwarf breeding in cotton and start a new race to determine the molecular regulatory mechanisms underlying dwarfism in cotton. We believe that proteomics can be used to unravel cotton’s physiological response under stress conditions toward crop improvement.

## Single-cell proteomics: A powerful futuristic tool to revolutionize cotton proteomics research

**Figure 3:**
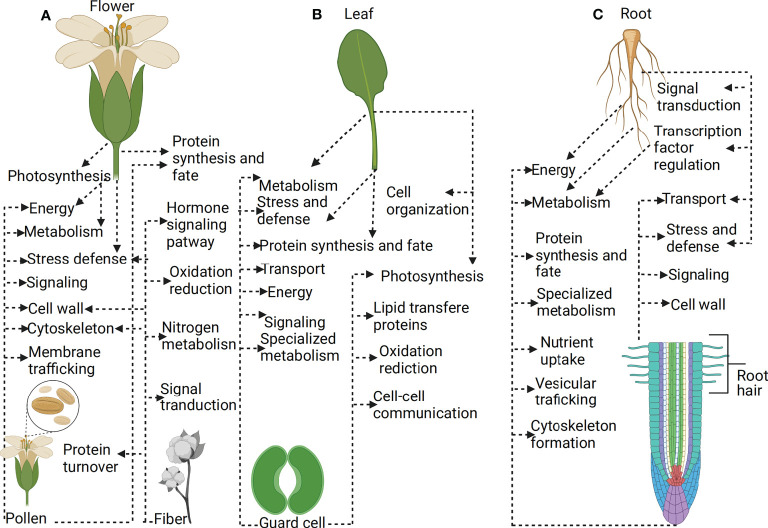
Plant single-cell type-proteomics. **(A)** Proteins expressed in flower versus protein expressed in the pollen **(B)** Proteins expressed in leaf versus protein expressed in the guard cell **(C)** Proteins expressed in root versus protein expressed in the root hair. Single-cell proteomics allows the identification of many proteins expressed within thousands of individual cells at a given time. Single-cell-type proteomics treats biological samples as heterogeneous, which reveals the actual function of cells in plant developmental processes or response to stress. The application of single cells proteomics has several advantages, such as providing specific information on cell function than the whole organ or tissue proteomics, which provide average information of cells.

Several biological processes involve the interaction of signal networks across a population of cells, organs, and whole tissues. Bulk-cell and tissue omics profiling technologies such as transcriptomics, proteomics, and metabolomics have been used to study cell type and gene expression in plant tissues. However, these bulk methods only generate the averages of cells, do not analyze a small number of cells, and cannot also provide heterogeneous cell information. Given that the heterogeneous cell information of individual cells can be obtained depending on the profiling method, single-cell expression profiling of plant tissues is the only holistic way of generating a deeper understanding of plant developmental processes or environmental adaptation (Dai and Chen, 2012; Clark et al., 2022). Proteomics of plant organs or tissues has uncovered several proteins in different plant cultivars during developmental changes or under varying environmental conditions (Baerenfaller et al., 2008; Hochholdinger et al., 2018; Liu Y. et al., 2019). However, several single-cell-type proteomics studies on cotton fiber, pollen grains, guard cells, and root hairs have proven to identify several important proteins ranging from defense, metabolism, signaling and transport, cytoskeleton, cell wall modification, lipid transfer, oxidation-reduction, among others, more than their mother tissue or organs such as a leaf, flower, and root (**Figure 3**). This is because single-cell-type proteomics does not treat the sample as a homogeneous sample but rather as a heterogeneous sample, which reveals the cells’ actual functions in biological processes. A number of single-cell-type proteomics studies involving pollen and fiber identified several proteins enriched in membrane trafficking, signal transduction, oxidation-reduction, N metabolism, cytoskeleton, cell wall modification, signaling, metabolism, stress defense, energy, protein synthesis and fate (Fernando, 2005; Dai et al., 2006; Petersen et al., 2006; Sheoran et al., 2007; Wu et al., 2008; Chen et al., 2009; Grobei et al., 2009; Pertl et al., 2009; Han et al., 2010; Hu et al., 2017; Zhou et al., 2019), while several organ/tissue studies involving flowers have demonstrated to be enriched in metabolism, stress and defense, photosynthesis, energy and protein synthesis and fate (Dafny-Yelin et al., 2005; Chua et al., 2010; Silveira and Carvalho, 2016) (**Figure 3A**). Also, different works of single-cell-type proteomics studies involving the guard cell have identified many proteins enriched in specialized metabolism, signaling, energy, transport, protein synthesis and fate, stress and defense, photosynthesis, lipid transfer, oxidation-reduction and cell-cell communication (Okamoto et al., 2004; Zhu et al., 2009; Lawrence et al., 2020; Balmant et al., 2021), while organ and tissue studies involving the leaf have demonstrated to be enriched in photosynthesis, cell organization, metabolism, stress and defense, and protein synthesis and fate (Wan and Liu, 2008; Khodadadi et al., 2017) (**Figure 3B**). In addition to those mentioned above, single-cell-type proteomics of root hair has identified several important proteins enriched in specialized metabolism, metabolism, synthesis and fate, energy, cell wall modification, signaling, stress and defense, and transport (Brechenmacher et al., 2008; Nestler et al., 2011; Brechenmacher et al., 2012), while organ/tissue studies involving roots have demonstrated to be enriched in transport mechanisms, energy, synthesis activity, signal transduction, transcription regulation, stress, and defense (Sun et al., 2017) (**Figure 3C**). Together, these studies enhanced our understanding of the role of particular proteins in cellular development, which highlighted insights into the molecular networks underlying the role of a particular type of plant cell and, to a large extent, revealed the significant difference between the proteomics of whole tissue or organ and single-cell-type proteomics.

## Conclusions and perspectives

Yield reduction in agricultural crops due to biotic and abiotic stress calls for understanding how plants respond to these stresses. In this post-genomic era, the integration of proteomics into the field of crop science will enrich genome annotation efforts and push forward the development of crop models for the elucidation of gene function influencing phenotypes for the success of field crops. Thus, studies involving cotton’s response to biotic and abiotic stresses at the proteome level have significantly contributed to our understanding of the molecular mechanisms underlying these responses. These studies have contributed to unraveling specific resistance and response traits displayed by plants under stress conditions. The proteins identified via proteomics analysis can further be investigated to finally assess their role in plant resistance processes, thus facilitating the efforts to develop stress-tolerant crops. Cotton proteomics enables the identification of key protein types responsible for a biological process under specific conditions in a particular tissue. Cotton proteomics also provides one of the best options for understanding the gene function and phenotypic changes during cotton fiber development and stress response, thus providing novel clues to guide further investigations and genetic improvement for high-quality cotton fiber. The past years have seen tremendous progress in studying low-abundance membrane proteins, leading to the development of different proteomics techniques. Meanwhile, further advances in proteomics technologies are required for higher precision. Considering the diverse and increasing number of recent single-cell proteomics studies reported (Clark et al., 2022; Potts et al., 2022), we believe that the application of high-throughput proteomics technology, such as single-cell proteomics, will provide a better understanding of the mechanisms surrounding cotton stress tolerance.

## Author contributions

Conceptualization of the project: XS; writing of the first draft: GB and XS; literature revision: YZ, SF, QM, and DTT; supervision and validation: XS; all authors contributed to the article and approved the submitted version.

## Funding

This research was supported by the National Natural Science Foundation of China (31670233).

## Conflict of interest

The authors declare that the research was conducted in the absence of any commercial or financial relationships that could be construed as a potential conflict of interest.

## Publisher’s note

All claims expressed in this article are solely those of the authors and do not necessarily represent those of their affiliated organizations, or those of the publisher, the editors and the reviewers. Any product that may be evaluated in this article, or claim that may be made by its manufacturer, is not guaranteed or endorsed by the publisher.
